# Epidemiology of *Staphylococcus aureus* in a burn unit of a tertiary care center in Ghana

**DOI:** 10.1371/journal.pone.0181072

**Published:** 2017-07-13

**Authors:** Nana Ama Amissah, Lieke van Dam, Anthony Ablordey, Opoku-Ware Ampomah, Isaac Prah, Caitlin S. Tetteh, Tjip S. van der Werf, Alexander W. Friedrich, John W. Rossen, Jan Maarten van Dijl, Ymkje Stienstra

**Affiliations:** 1 Department of Internal Medicine/Infectious Diseases, University of Groningen, University Medical Center Groningen, Groningen, the Netherlands; 2 Department of Bacteriology, Noguchi Memorial Institute for Medical Research, University of Ghana, Legon, Ghana; 3 Department of Medical Microbiology, University of Groningen, University Medical Center Groningen, Groningen, the Netherlands; 4 The Reconstructive Plastic Surgery and Burn Center, Korle Bu Teaching Hospital, Korle Bu, Ghana; Rockefeller University, UNITED STATES

## Abstract

**Background:**

In developing countries, hospitalized burn victims are at high risk of nosocomial infections caused by *Staphylococcus aureus*. Risk factors include poor infection control practices, prolonged hospitalisation and limited capacity for laboratory microbiological analyses. These problems are compounded by widespread use of antibiotics that drives the spread of multi-drug resistant bacteria.

**Methods:**

During the study period (November 2014-June 2015), nasal and invasive *S*. *aureus* isolates were collected consecutively from patients and healthcare workers (HCWs) within the burn unit of the Reconstructive Plastic Surgery and Burn Center of Korle Bu Teaching Hospital in Ghana. Antibiotic prescription, antibiotic susceptibility and bacterial typing were used to assess antibiotic pressure, antibiotic resistance, and possible transmission events among patients and HCWs.

**Results:**

Eighty *S*. *aureus* isolates were obtained from 37 of the 62 included burn patients and 13 of the 29 HCWs. At admission, 50% of patients carried or were infected with *S*. *aureus* including methicillin resistant *S*. *aureus* (MRSA). Antibiotic use per 100 days of hospitalization was high (91.2 days), indicating high selective pressure for resistant pathogens. MRSA isolates obtained from 11 patients and one HCW belonged to the same *spa*-type t928 and multi-locus sequence type 250, implying possible transmission events. A mortality rate of 24% was recorded over the time of admission in the burn unit.

**Conclusion:**

This study revealed a high potential for MRSA outbreaks and emergence of resistant pathogens amongst burn patients due to lack of patient screening and extended empirical use of antibiotics. Our observations underscore the need to implement a system of antibiotic stewardship and infection prevention where microbiological diagnostics results are made available to physicians for timely and appropriate patient treatment.

## Introduction

Burns are a major public health problem globally, but this problem is especially grave in low- and middle-income countries (LMIC). Burns in LMIC lead to high mortality rates of over 95% and the survivors frequently suffer from life long disabilities [1].

In sub-Saharan Africa, prevention and management of burns receive limited attention. The general public lacks knowledge on prevention and first aid [2,3], and 84% of burns occur in children below the age of ten years [4]. The few burn units available are frequently inadequately equipped and lack the needed multidisciplinary approach, including infection control programs [5–8]. Burns in sub-Saharan Africa are often related to the use of petroleum products, leading to an equal ratio of scalds to flame burns [4,9]. This is exemplified by a petroleum fire disaster in Ghana in 2015, which claimed about 200 lives in the southeastern part of the country (daily graphic, 2^nd^ June 2015). Reliable data on burn victims’ mortality is difficult to obtain since people may die on the spot or when discharged from the hospital, and mortality rates cannot be interpreted without data on the Total Body Surface Affected (TBSA). A recent study, described a mortality rate of 13% in the burn patients who visited a university hospital in the middle part of Ghana [10].

Infections are common in burn patients and, in particular, invasive infections are responsible for 28–65% of burn deaths worldwide [11,12]. *Staphylococcus aureus* is an opportunistic pathogen that causes skin and soft tissue infections as well as invasive infection in burn patients. In a South African intensive care burn unit, methicillin resistant *S*. *aureus* (MRSA) was the third most common organism encountered in blood cultures. In this setting 17% of the patients with MRSA-positive blood cultures died [13]. In addition, invasive infections caused by *Acinetobacter baumannii* and *Pseudomonas aeruginosa* were found to frequently lead to death of burn patients.

Recently, studies on *S*. *aureus* genetic diversity in healthcare and community settings have detected *spa*-types t084, t314 and t355 to be predominant in Ghana [14–18]. However, there has so far been no data published on the prevalence of *S*. *aureus* and this pathogen’s association with invasive infections in burn centers in Ghana. The present study was therefore aimed at assessing the epidemiology of *S*. *aureus* in burn patients in the Reconstructive Plastic Surgery and Burn Center of the Korle Bu Teaching Hospital (KBTH), Korle Bu, Ghana, during a seven-month survey. Burn patients and healthcare workers (HCWs) were screened for *S*. *aureus*, and the antibiotic resistance profiles of nasal or invasive *S*. *aureus* isolates were investigated. Data from this study is intended to guide future antimicrobial therapy and effective hospital infection control.

## Materials and methods

The burn unit of the Reconstructive Plastic Surgery and Burn Center of KBTH records about 328 admissions per year with about 27% in-hospital mortality. All burn patients are referred from either the intensive care unit (ICU) of the hospital or from other regional or district hospitals in the country. Patients are referred based on the severity of the burns, the surgical intervention needed, and sometimes for logistical reasons. The burn unit comprises six beds each in the adult male and female wards, seven beds in the children’s ward and two beds in the isolation ward. The unit has an operating theater and a treatment/dressing room. Wounds of burn patients are cleaned with water containing antiseptic solution (cetrimide 3% w/v and chlorhexidine gluconate 0.3% w/v). The wounds are subsequently rinsed with normal saline and topical ointments containing antiseptics. Silver sulphadiazine and sulphadiazine are applied onto the burn wound. This is followed by an occlusive dressing with paraffin-soaked gauze, sterile cotton and a bandage. The dressings are changed every 72 hours.

### Study participants

Following ethical clearance from the ethics committee of the Noguchi Memorial Institute for Medical Research (FEDERAL WIDE ASSURANCE FWA 00001824), patients’ and HCWs data and samples were collected for the present study. All samples were collected upon written informed consent or assent from all participants aged ≥12 years, and consent from a parent, caretaker, or legal representative of any participant below the age of 18 years.

All patients consecutively admitted to the burn unit of the Reconstructive Plastic Surgery and Burn Center of KBTH, Ghana, from November 2014 to May 2015 were invited to participate. General patient information including age, gender, type of injury, length of stay, outcome (death or discharge), TBSA and use of antibiotics were retrieved from the patients’ files. However, patients’ data from the referral centers prior to admission in this burn unit was unavailable. Antibiotic consumption was expressed as the total number of days of antimicrobial use per total number of admission days.

Specifically, both nares and wounds were swabbed using dry eSwabs, which were subsequently suspended in 1 ml liquid Amies medium (Copan Diagnostics Inc., Murrieta, CA, USA). Blood cultures were collected from the patients upon admission in the burn unit and biweekly during the change of wound dressings. Blood cultures testing positive for *S*. *aureus*, or MRSA carriage were reported to the responsible clinician to ensure appropriate clinical management. Nasal swabs were collected from healthcare workers once per two months for the duration of the seven months study period.

### Bacterial isolation, identification, *mec*A detection and antimicrobial susceptibility testing

All swabs were streaked on 5% sheep blood agar (BA) and the respective plates were incubated at 37˚C overnight. Presumptive *S*. *aureus* colonies were tested for coagulase using the Pastorex Staph Plus test (Bio-rad, Marnes-la-Coquette, France) and *nuc* PCR [19]. *S*. *aureus* isolates were confirmed using matrix-assisted laser desorption ionization-time of flight mass spectrometry (MALDI-TOF MS) with a microflex LT Biotyper (Bruker Daltonics, Bremen, Germany) according to manufacturer’s instructions.

Blood cultures collected biweekly from patients were incubated in the BACTEC-9240 blood culture system (Becton Dickinson, Sparks, MD, USA) for seven days. Cultures were made from blood samples that gave positive signals for identification of *S*. *aureus* as described previously. To identify MRSA, all *S*. *aureus* isolates were screened for *mec*A gene as previously described [20]. Antimicrobial susceptibility testing of *S*. *aureus* was initially performed using the disk diffusion method on Muller Hinton Agar and interpreted according to the CLSI guidelines at Noguchi Memorial Institute for Medical Research in Ghana [21]. These analyses were, however, only performed on the *S*. *aureus* isolates from the nares and wounds of patients and/or HCWs that tested positive for the *mec*A gene, and for the methicillin sensitive *S*. *aureus* (MSSA) and MRSA isolates from invasive infections. Antimicrobial susceptibility testing was further performed on all *S*. *aureus* isolates with the VITEK 2 system (AST-P633, bioMerieux Corporate, Marcy l’Etoile, France) according to the manufacturer’s instructions at the diagnostics laboratory of the University Medical Center Groningen in the Netherlands. The used cards contained the following antibiotics: benzylpenicillin, cefoxitin, chloramphenicol, ciprofloxacin, clindamycin, erythromycin, fosfomycin, fusidic acid, gentamicin, kanamycin, linezolid, mupirocin, oxacillin, rifampicin, teicoplanin, tetracycline, tobramycin, trimethoprim/sulfamethoxazole and vancomycin. The minimum inhibitory concentration results were interpreted according to EUCAST guidelines (www.eucast.org).

### Detection of the Panton-Valentine leukocidin genes and bacterial typing

All *S*. *aureus* isolates were screened for the presence of Panton Valentine Leucocidin (PVL) by PCR as previously described [22]. *Spa*-typing of *S*. *aureus* isolates was performed as previously described by Harmsen *et al*. [23]. DNA sequences were determined using an ABI Prism 3130 genetic analyser (Applied Biosystems, Foster City, USA). *Spa*-types were assigned using the Ridom Staph Type software version 2.2.1 (Ridom GmbH, Würzburg, Germany) [23]. Multilocus Sequence Typing (MLST) was performed on a subset of isolates (n = 70) as described by Enright *et al*. [24].

### Statistical analysis

Descriptive statistics including median and inter-quartile range (25–75%) were used for quantitative data (age, TBSA of the burn injury, and length of hospital stay). The association between patients’ characteristics and the carriage of *S*. *aureus* was calculated using Pearson chi-square or Fisher’s exact test and Mann-Whitney U test as appropriate. Statistical analysis was performed with SPSS 22.0 (SPSS Inc., Chicago, IL) software.

## Results

### Patient characteristics

Sixty-seven patients were admitted to the burn unit during the study period. Sixty-two patients were recruited while five patients declined participation. The patients were referred from 20 hospitals (n = 26) in Ghana and the ICU of the KBTH (n = 36). General patient characteristics are presented in Table 1. The median age was 25 years (IQR: 3–35) and 36% of the participants were younger than 10 years. Flames accounted for the cause of burns for the majority of the patients. The median TBSA of the burn was 15% (IQR: 7.8–28.2%).

**Table 1:** Patient characteristics.

Patient characteristics	Number (IQR or % frequency)
Sex (female, percentage)	37 (60%)
Age (years, median (IQR))	25 (3–35)
Type of burn	
Acid	1 (1.6%)
Chemicals	2 (3.2%)
Electrical	1 (1.6%)
Flame	24 (38.7%)
Gas	16 (25.8%)
Scald (hot fluids)	18 (29%)
TBSA (median %)	15 (7.8–28.2)
Hospital stay (days)*	11 (5–17.5)
In-hospital mortality	15 (24.2%)

*Hospital stay refers exclusively to hospitalization in the burn unit

Twenty-nine HCWs (resident (n = 1), house officer (n = 1), nurses (n = 30) and cleaners (n = 2)) were included during the study period. Twenty-four HCWs completed the study; the other HCWs were transferred to other units within the hospital. Twenty-nine HCWs were swabbed on each occasion including newly transferred HCWs. None of the HCWs declined participation during the study.

### *S*. *aureus* epidemiology in the burn unit

Eighty *S*. *aureus* isolates were obtained from 37 (60%) of the 62 patients and 13 (45%) of the 29 HCWs. Twenty-two (28%) isolates tested positive for the *mecA* gene. On the first day of admission (within 24 hours) 31 (50%) patients carried at least one *S*. *aureus* strain in their nares (n = 21), burn wounds (n = 17), or both (n = 9). Four patients had *S*. *aureus* detected in their blood culture. Two of these patients carried *S*. *aureus* in their nares and burn wounds simultaneously. Seven (11%) of the 62 patients tested MRSA-positive on the first day of admission, two of whom had MRSA detected in their blood cultures. Four of these seven MRSA-positive patients were referred from the KBTH ICU and three from one of the other referral centers. In eight (13%) of the 62 patients *S*. *aureus* was cultured after the admission. These eight patients carried at least one strain in their nares (n = 5), burn wounds (n = 5) or both (n = 3). In five of these eight patients the acquired *S*. *aureus* strain was MRSA.

Thirteen (45%) of the 29 HCWs tested positive at least once with *S*. *aureus* during the study period. One of the HCWs carried MRSA.

### Antibiotic prescription and *S*. *aureus* resistance pattern

Cefuroxime was one of the antibiotics frequently consumed by burn patients. Further use of antimicrobials was triggered by clinical events (fever or suspected sepsis) or—in a minority of cases—by the detection of *S*. *aureus* invasive infections and sometimes MRSA wound colonization during the current study. The antibiotics prescribed in the burn unit during the study period are presented in Table 2.

**Table 2:** Number of days of antibiotics prescribed per 100 days in the burn unit and antibiotic resistances of the 80 investigated *S*. *aureus* isolates.

Antibiotics	Number of days of antibiotic used per 100 in-patient hospital days	No. (resistance rate %)
Amoxicillin-clavulanic acid	1.9	**-**
Cefuroxime	60.6	**-**
Ceftazidime	1.6	**-**
Benzylpenicillin	-	80 (100)
Oxacillin	-	22 (28)
Vancomycin	0.4	0 (0)
Gentamicin	11.9	21 (26)
Tobramycin	-	21 (26)
Kanamycin	-	22 (28)
Ciprofloxacin	5.6	21 (26)
Levofloxacin	4.1	-
Chloramphenicol	-	35 (44)
Clindamycin	-	1 (1)
Erythromycin	2.3	1 (1)
Fosfomycin	-	0 (0)
Fusidic acid	-	0 (0)
Mupirocin	-	0 (0)
Linezolid	-	0 (0)
Teicoplanin	-	0 (0)
Rifampicin	-	1 (1)
Tetracycline	-	41 (51)
Trimethoprim/sulfamethoxazole	-	20 (25)
Metronidazole	2.9	-
**Total**	91.2	

“-” indicates that antibiotics were not prescribed to burn patients or that the resistance of *S*. *aureus* to the respective antibiotics was not tested.

All 80 *S*. *aureus* isolates were tested for susceptibility to antibiotics. The results are presented in Table 2. None of the isolates were resistant to fosfomycin, fusidic acid, linezolid, mupirocin, teicoplanin, or vancomycin. On the other hand, all isolates were resistant to benzylpenicillin and over half of the isolates (51%) were resistant to tetracycline. Twenty-two (28%) isolates were resistant to cefoxitin and oxacillin, and 21 of these isolates from 11 patients and one HCW displayed a similar antibiotic resistance pattern to gentamicin, kanamycin, tobramycin and ciprofloxacin (S1 Table).

### Detection of PVL genes and bacterial-typing

PVL-encoding genes were detected in 27 (34%) of the 80 isolates obtained from 17 (27%) of the 62 patients (anterior nares [n = 6] and wound [n = 12]) and 6 (21%) of the 29 HCWs. *S*. *aureus* isolates from patients and HCWs were assigned 20 different *spa*-types and 16 STs including four new STs: ST3248, ST3249, ST3250, ST3251 and one untypeable isolate (Table 3, S1 Table). *S*. *aureus* with the *spa*-types t008, t084, t127, t2055, t304, t311, t5132, t645, t827, t8453 (MSSA) and t928 (MRSA) were obtained from the nares of patients. *S*. *aureus* obtained from wound swabs of patients belonged to *spa*-types t084, t311, t314, t355, t537, t645, and t939 (MSSA), and t008 and t928 (MRSA) (S1 Table). In the case of five patients, this may suggest autoinoculation from the nares to wound or *vice versa*. The *spa*-types t127, t355, t861, t8860 (MSSA) and t928 (MRSA) were identified in *S*. *aureus* invasive infections. *S*. *aureus* carriage by HCWs was identified as MSSA (t008, t024, t084, t127, t1816, t861 and t963) and MRSA (t928), and most HCWs carried *S*. *aureus* with the same genotype on more than one occasion. Interestingly, 21 of the 22 MRSA isolates belonged to the *spa*-type t928 (ST250) while the other MRSA isolate belonged to *spa*-type t008 (ST8). The MRSA isolates with *spa*-type t928 were cultured from 11 patients and one HCW.

**Table 3:** Antibiotic resistance profiles and detection of PVL genes in *S*. *aureus* isolates with different *spa*-types.

*Spa*-type	ST	No. of isolates	Major antibiograms	PVL-positive
t008	8, 2021	5	Ben	0
t024	8	1	Ben	0
t084	15, 3249, -	10	Ben, tri/sulf	7
t127	1, 3248	5	Ben	0
t1816	15	3	Ben, tri/sulf	2
t2055	2434	1	Ben, tet, chl, tri/sulf	0
t304	6	1	Ben	0
t311	5	2	Ben, tet, chl, rif, tri/sulf	0
t314	121	2	Ben, tet, tri/sulf	2
t355	152	8	Ben, chl	7
t5132	508	1	Ben	1
t537	3251	5	Ben, tet	3
t645	3250	4	Ben	4
t827	508	2	Ben, tri/sulf	0
t8453	508	1	Ben, tri/sulf	0
t861	508	3	Ben	0
t8860	45	1	Ben	0
t928	250	21	Ben, oxa, gen, kan, tob, cip, tet, chl	0
t939	45	1	Ben	0
t963	15	3	Ben	1

Ben- benzylpenicillin, oxa- oxacillin, gen- gentamicin, kan- kanamycin, tob- tobramycin, cip- ciprofloxacin, tet- tetracycline, chl- chloramphenicol, rif- rifampicin, tri/sulf- Trimethoprim/sulfamethoxazole. The “-” indicates that an isolate was untypeable by MLST.

### *S*. *aureus* invasive infection

*S*. *aureus* was detected in the blood cultures during the biweekly routine samplings in six (10%) of the 62 patients hospitalized. On the first day of admission, 4 (6%) patients already had blood cultures positive with *S*. *aureus*. Seven *S*. *aureus* isolates were obtained from the blood cultures of the six patients. Three of these isolates were MRSA (t928), four were MSSA (t127, t355, t861 and t8860) and all 7 isolates were PVL-negative (S1 Table). Physicians received the results including the antibiotic susceptibility pattern to treat patients for invasive infection. Patients with MRSA invasive infection were treated by the attending physicians with either erythromycin or vancomycin while patients with MSSA invasive infection were treated with erythromycin, gentamicin or ciprofloxacin. However, the therapy was sometimes changed from vancomycin to erythromycin, because patients could not afford vancomycin treatment.

### In-hospital mortality

A mortality rate of 24% during the admission at the burn unit was recorded. Half of the patients with a TBSA of 19% or more died during the admission (LD50 of 19%). Patients who died were in the hospital for a median of 9 days (IQR: 2–42). None of the patients with *S*. *aureus* detected in the blood culture died (RR 0.23; 0.01–3.87). Mortality in patients with *S*. *aureus* cultured at least once from wounds, blood or nares during the admission was 18.9%, whereas mortality in patients without *S*. *aureus* cultured during their admission was 32% (RR 0.59; 0.25–1.42) (Table 4).

**Table 4:** Carriage of or infection with *S*. *aureus* at least once during admission of patients.

	*S*. *aureus* at least once (positive)	No *S*. *aureus*	p-value
Age (years)	26	25	0.57*
TBSA (median %)	15	16.5	0.83*
Sex: female (%)	18 (66%)	19 (54%)	0.32^#^
Mortality rate	5 (18.5%)	10 (28%)	(RR 0.59; 0.25–1.42)

*, Mann-Whitney U Test used for analysis

^#^, Pearson, chi-square used for analysis

RR, relative risk

## Discussion

The present study describes the *S*. *aureus* epidemiology in burn patients and HCWs in a burn unit in Ghana. To our knowledge, this is the first report of such data from Ghana. In developed countries, the elderly are often susceptible to burn injuries [25,26]. Our study describes a much younger patient group with burns, which is consistent with reports from other health centers in Ghana and other African countries, where children of 10 years and below represent the majority of burn victims [4,10]. Most of the burn patients suffered from flame burns (39%) due to the use of candles and kerosene lamps for lighting as a result of the national electricity crisis in Ghana.

Referrals from the 20 hospitals and the ICU of the KBTH were often made due to the worsening conditions of patients, lack of resources or skilled professionals for specialized care of third degree burns, or logistical reasons. Routine screening of patients for MRSA carriage or multi-drug resistant organisms (MDRO) are not performed in the burn unit. Here, infection control measures were taken with regard to only one MRSA patient, which indicates the high risk of patient-to-patient transmission events. Interestingly, the MRSA cases included patients who tested positive on the day of admission and patients who acquired the MRSA during admission. The majority of these isolates (21 of 22) were shown to belong to the *spa*-type t928 and ST250. Thus, patients that tested positive on admission could be the possible source of the nosocomial MRSA transmission events. This observation highlights the need for implementing infection control and prevention measures in referring health care centers to prevent outbreaks of disease that currently remain unnoticed. Previous studies have shown that colonized but non-isolated patients increase the risk for nosocomial transmission events [27,28]. Since most presently identified MRSA cases were referrals, it is important that patients from referral centers that frequently report MRSA, such as the KBTH ICU, be screened for MRSA when transferred to the burn unit to prevent possible outbreaks. Further, almost half of the HCWs (45%) were transient carriers of *S*. *aureus*, including MRSA, which indicates their possible involvement in nosocomial transmission events in the burn unit. *S*. *aureus* carriage of 23% among HCWs in other departments in this hospital has been reported [29]. In this respect, it should be noted that bacterial typing methods, such as *spa*-typing and MLST, are not optimal for inferring nosocomial events, because they have a relatively low discriminatory power compared to whole genome sequencing. Therefore, whole genome sequencing analyses of the MRSA isolates (ST250) will be needed to confirm such events.

The high incidence of MRSA isolates with ST250 is noteworthy. First reported as the cause of epidemic MRSA disease and dominant MRSA genotype in the mid-1960s in Australia, Europe (Denmark, Germany, Switzerland), Uganda and the United Kingdom, and during the 1970s-1980s in Ireland [30–32], there are presently no reports of ST250-I MRSA in these regions [31]. Recently, ST250-I MRSA has, however, emerged in skin and soft tissue infections in a health care center in Ghana [14]. Further, our present data shows a wider distribution of ST250-MRSA with the SCC*mec* type IV variant from other referral health care centers in Ghana suggesting the rapid dissemination of this strain in hospitals of this country.

The overall use of antibiotics per 100 in-patient hospital days was observed to be high due to the extended use of empirical treatment and lack of microbiological diagnostic results. Clinical isolates obtained from burn patients have been reported to be resistant to commonly prescribed drugs in burn units [33–35]. Thus, appropriate prescription of antibiotics is important to prevent emergence of resistant bacteria. However, this can only be achieved if the burn units are equipped with some baseline epidemiological and microbiological data.

We observed a 27% of carriage of PVL-positive *S*. *aureus* in burn patients. PVL-positive MRSA was not detected in this study. PVL-positive MRSA has been reported in burn centers in the United Kingdom (9%), United States of America (2.5%) and Iran (7%) [36–38]. Additionally, none of the *S*. *aureus* isolates from invasive infections carried the PVL gene. This is remarkable as, more frequently, *S*. *aureus* isolates from invasive infections have been reported to carry PVL-encoding genes in Ghana and the Democratic Republic of the Congo (49–75%) [18,39]. Here, it should be mentioned that the major *S*. *aureus spa*-types t084, t355 and t928 as identified in our study have been described previously in Ghana [14–18].

The mortality rate (24%) recorded in our study was within the range (9–27%) reported by burn centers in other sub-Saharan African countries [4]. We observed 100% mortality in patients with TBSA burns greater than 40% [40,41]. The lethal Dose 50 (the percent TBSA at which 50% of patient survive) is a measure of the quality of care given to burn patients. In the present study, half of the patients with a TBSA of 19% died during the admission, indicating that burns with such a relatively small size lead to poor clinical outcomes in Ghana. This contrasts with settings in more affluent countries, where therapeutic advancement in burn care has increased the LD50 to 25–77% [25,42,43].

The lower mortality rate observed in patients carrying and/or infected with *S*. *aureus* was a surprising finding. However, this may be explained by the accessibility of the microbiological diagnostic results and timely and appropriate treatment of patients during the study period. Further diagnostics on bacteria other than *S*. *aureus* were not performed. This lack of screening for microorganisms other than *S*. *aureus* that nonetheless are commonly involved in infectious complications of burn wounds is a limitation to this study. Gram-negative bacteria such as *Pseudomonas aeruginosa* and *Acinetobacter baumannii* have been reported to frequently cause nosocomial infections in burn patients and may have antimicrobial resistance patterns severely limiting the treatment options in this health care setting [44–47]. Of note, hospital hygiene measures in the burn unit were not assessed in the present study. Previous studies have reported low compliance of hand hygiene by health personnel [48–50] and high prevalence of pathogenic bacteria on surfaces of operating theaters, door handles, desktops and lavatories in health care settings in Ghana [51].

In conclusion, our study shows that it is very well possible that MRSA outbreaks occur unnoticed in the presently investigated burn unit. This is highly worrisome as this burn unit is representative for the situation in many sub-Saharan burn units. Although this has not become directly evident from our study, improving the capacity for microbiological diagnostics and antibiotic stewardship will most likely reduce the mortality rate in such burn units by optimizing the use of antimicrobials and earlier detection of outbreaks of highly drug resistant pathogens.

## Supporting information

S1 Table *S*. *aureus* isolates from burn patients and healthcare workers. “-” and empty cells under the ST column indicate that isolate was untypable and MLST was not performed for these isolates respectively.
(XLSX)
